# Assessing the Use of German Claims Data Vocabularies for Research in the Observational Medical Outcomes Partnership Common Data Model: Development and Evaluation Study

**DOI:** 10.2196/47959

**Published:** 2023-11-07

**Authors:** Elisa Henke, Michéle Zoch, Michael Kallfelz, Thomas Ruhnke, Liz Annika Leutner, Melissa Spoden, Christian Günster, Martin Sedlmayr, Franziska Bathelt

**Affiliations:** 1 Institute for Medical Informatics and Biometry, Carl Gustav Carus Faculty of Medicine, Technische Universität Dresden, Dresden, Germany; 2 Odysseus Data Services GmbH, Berlin, Germany; 3 Wissenschaftliches Institut der AOK (AOK Research Institute), Berlin, Germany; 4 Thiem-Research GmbH, Cottbus, Germany

**Keywords:** OMOP CDM, interoperability, vocabularies, claims data, OHDSI, Observational Medical Outcomes Partnership, common data model, Observational Health Data Sciences and Informatics

## Abstract

**Background:**

National classifications and terminologies already routinely used for documentation within patient care settings enable the unambiguous representation of clinical information. However, the diversity of different vocabularies across health care institutions and countries is a barrier to achieving semantic interoperability and exchanging data across sites. The Observational Medical Outcomes Partnership (OMOP) Common Data Model (CDM) enables the standardization of structure and medical terminology. It allows the mapping of national vocabularies into so-called standard concepts, representing normative expressions for international analyses and research. Within our project “Hybrid Quality Indicators Using Machine Learning Methods” (Hybrid-QI), we aim to harmonize source codes used in German claims data vocabularies that are currently unavailable in the OMOP CDM.

**Objective:**

This study aims to increase the coverage of German vocabularies in the OMOP CDM. We aim to completely transform the source codes used in German claims data into the OMOP CDM without data loss and make German claims data usable for OMOP CDM–based research.

**Methods:**

To prepare the missing German vocabularies for the OMOP CDM, we defined a vocabulary preparation approach consisting of the identification of all codes of the corresponding vocabularies, their assembly into machine-readable tables, and the translation of German designations into English. Furthermore, we used 2 proposed approaches for OMOP-compliant vocabulary preparation: the mapping to standard concepts using the Observational Health Data Sciences and Informatics (OHDSI) tool Usagi and the preparation of new 2-billion concepts (ie, *concept_id* >2 billion). Finally, we evaluated the prepared vocabularies regarding completeness and correctness using synthetic German claims data and calculated the coverage of German claims data vocabularies in the OMOP CDM.

**Results:**

Our vocabulary preparation approach was able to map 3 missing German vocabularies to standard concepts and prepare 8 vocabularies as new 2-billion concepts. The completeness evaluation showed that the prepared vocabularies cover 44.3% (3288/7417) of the source codes contained in German claims data. The correctness evaluation revealed that the specified validity periods in the OMOP CDM are compliant for the majority (705,531/706,032, 99.9%) of source codes and associated dates in German claims data. The calculation of the vocabulary coverage showed a noticeable decrease of missing vocabularies from 55% (11/20) to 10% (2/20) due to our preparation approach.

**Conclusions:**

By preparing 10 vocabularies, we showed that our approach is applicable to any type of vocabulary used in a source data set. The prepared vocabularies are currently limited to German vocabularies, which can only be used in national OMOP CDM research projects, because the mapping of new 2-billion concepts to standard concepts is missing. To participate in international OHDSI network studies with German claims data, future work is required to map the prepared 2-billion concepts to standard concepts.

## Introduction

### Background and Significance

**Figure 1.**
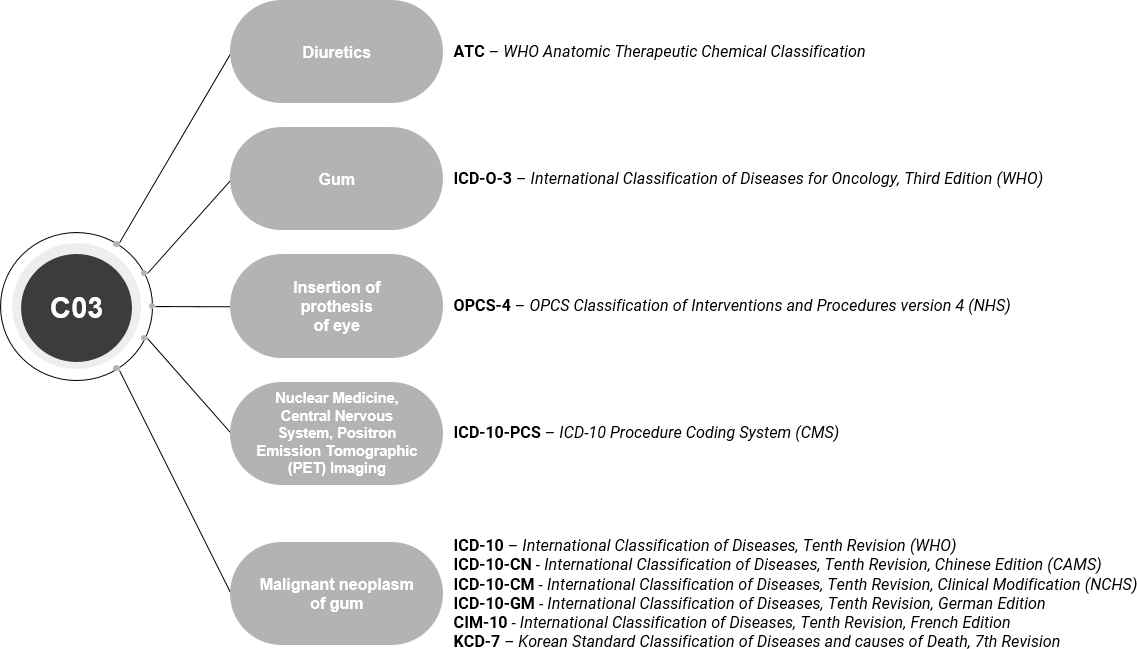
Overview of different meanings of the code C03 across various vocabularies. CAMS: Chinese Academy of Medical Sciences; CMS: Centers for Medicare & Medicaid Services; NCHS: National Center for Health Statistics; NHS: National Health Service; OPCS: Office of Population Censuses and Surveys; WHO: World Health Organization.

To generate reliable evidence in the health care sector, real-world data (RWD) can be used. RWD comprise observational data that are routinely collected in the context of patient care from various sources [1]. National classifications and terminologies enable the unambiguous representation of, for example, diagnoses (*International Classification of Diseases, Tenth Revision, German Edition* [*ICD-10-GM*]), procedures (*Operationen- und Prozedurenschlüssel* [Operations and Procedures Classification]), or laboratory data (Logical Observation Identifiers Names and Codes). Because these vocabularies are already routinely used for documentation within patient care settings, RWD already have a structured set of clinical information. However, each health care institution and country can have their own classifications, terminologies, or internally used set of codes. The diversity of different vocabularies is a barrier to achieving semantic interoperability and exchanging data across health care institutions and countries, as exemplarily shown in Figure 1. The code “C03” has 5 different semantic meanings covering the domains of drug, anatomic site, procedure, and condition [2]. Conducting research based on local vocabularies, terminologies, or classifications would result in custom analysis scripts for each site involved in a study. This not only entails high maintenance and time costs but is also unsustainable.

As a prerequisite for using data from heterogeneous data sources for international research and thus preventing the development of individual analysis scripts, harmonization and transformation to a common data model (CDM) are required. In recent years, the Observational Medical Outcomes Partnership (OMOP) CDM fostered by the Observational Health Data Sciences and Informatics (OHDSI) has become an essential open community data standard for research with RWD [3,4]. The OMOP CDM combines standardized data tables with centrally provided standardized vocabularies to ensure syntactic and semantic interoperability. The standardized vocabularies are represented in the OMOP CDM through concepts that enable the unique identification of all clinical events in the OMOP CDM. The concepts in the OMOP CDM are divided into standard and nonstandard concepts. Standard concepts provide normative expressions for international analyses and research based on the OMOP CDM. As an example, the Standard Nomenclature of Medicine concepts are mostly standard concepts in the OMOP CDM, for example, for the *Condition* domain. In contrast, nonstandard concepts are used to store codes of national vocabularies often used in source data, such as *ICD-10-GM*. The conversion (“mapping”) of nonstandard to standard concepts is part of OMOP CDM vocabulary tables and represented as concept relationships. Similar to the concepts themselves, the mapping is provided through the central OHDSI vocabularies repository Athena [5]. The advantage of standardized OMOP tables is that the source values and associated concepts, as well as the standardized concepts, are retained. Thus, it is always clear (1) what was part of the source data and (2) which concepts can be used for international research.

The main challenge faced by many researchers is the mapping of local source codes to OMOP CDM standard concepts [6-9]. Within our project “Hybrid Quality Indicators Using Machine Learning Methods” (Hybrid-QI) [10], we face this challenge during the transformation of German clinical data and claims data into the OMOP CDM. The aim of the project is the linkage of German clinical data and claims data into the OMOP CDM, to increase the effectiveness of quality measurement based on risk-adjusted quality indicators. To enable research based on 2 different heterogeneous data sets, the OMOP CDM is used for data harmonization. Although we have already successfully completed the semantic mapping of our clinical data [11], the mapping of German claims data comprising inpatient and outpatient data is still an open issue. To address this challenge, we focus first on the vocabularies used in German claims data as they build the basis for further semantic mapping to the OMOP CDM.

### State of the Art

In an initial analysis of the current coverage of German claims data vocabularies in the OMOP CDM by Henke et al [12], it was shown that 55% (11/20) of the vocabularies are not available. Only 15% (3/20) of the vocabularies are currently present in Athena. The remaining 30% (6/20) of the vocabularies can be mapped to standard concepts in the OMOP CDM by using the *source_to_concept_map* table in the OMOP CDM. As a consequence, not all source codes used in German claims data can be represented in the OMOP CDM using standard concepts. Instead of losing the data during the transformation into the OMOP CDM, Sathappan et al [13] suggest storing the source codes in the *_source_value columns of the OMOP CDM and mapping them to the *concept_id* of 0. However, this would again lead to the problem of using source codes for research as described earlier. In particular, the lack of associated nonstandard concepts in the OMOP CDM results in a loss of information about the vocabulary originally used in the source.

To our knowledge, no one so far has tried to prepare the missing 55% (11/20) of the German vocabularies for the OMOP CDM.

### Objectives

The purpose of this paper is to increase the coverage of German vocabularies in the OMOP CDM. With our study, we want to make a major contribution to research with RWD based on the OMOP CDM by:

1. Completely transforming source codes used in German claims data to the OMOP CDM without data loss and
2. Making German claims data usable for research based on the OMOP CDM.

## Methods

### Ethical Considerations

Only synthesized claims data were used for our purposes, and therefore, no ethics approval was required.

### Study Data

The preparation of missing German vocabularies and its evaluation was done in the context of the Hybrid-QI project. The following four different medical indications were chosen as examples for the development of hybrid quality indicators:

1. Acute myocardial infarction
2. Cerebral infarction or intracerebral hemorrhage
3. Colorectal resection for carcioma
4. Shoulder endoprosthesis or osteosynthesis for proximal humerus fracture

**Figure 2.**
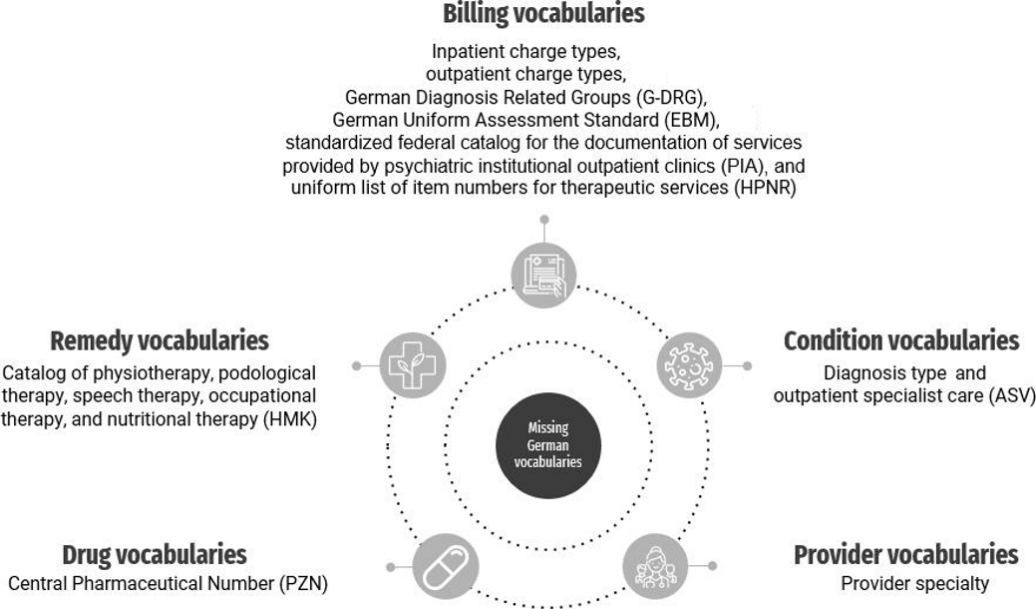
German vocabularies currently not available in the Observational Medical Outcomes Partnership (OMOP) Common Data Model (CDM). ASV: Ambulante spezialfachärztliche Versorgung; EBM: Einheitlicher Bewertungsmaßstab; HMK: Heilmittelkatalog; HPNR: Heilmittelpositionsnummern; PIA: Bundeseinheitlicher Katalog für die Dokumentation der Leistungen der psychiatrischen Institutsambulanzen; PZN: Pharmazentralnummer.

To provide source data for data harmonization in the OMOP CDM, we used synthesized claims data from the German local health care funds (Allgemeine Ortskrankenkassen), which are based on real data. This data set includes billing data from 10,000 patients for a 6-year period comprising 558 MB of tabular data. For our purpose, we only focused on the source codes of the missing German vocabularies and their associated documentation or billing date. The German vocabularies considered in the following steps for the preparation in the OMOP CDM are summarized in Figure 2. We categorized the vocabularies according to their definitions and codes as remedy, billing, drug, condition, and provider vocabularies.

### Vocabulary Preparation Approach

#### Overview

**Figure 3.**
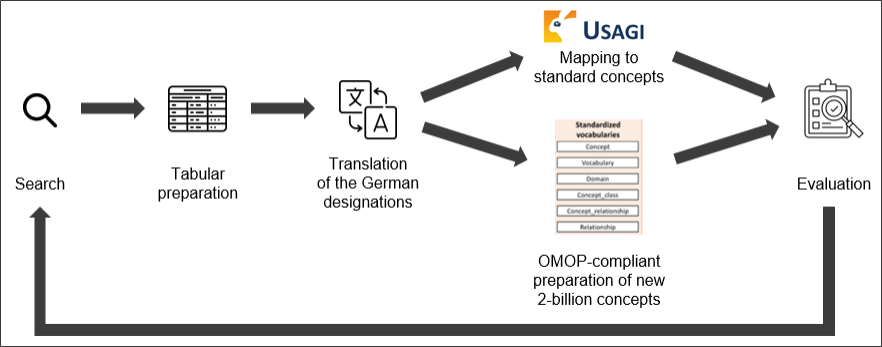
Vocabulary preparation approach. OMOP: Observational Medical Outcomes Partnership.

To prepare missing German vocabularies for the OMOP CDM, we defined an approach divided into 5 main steps (see Figure 3). First, we performed a selective search to identify all codes of the corresponding vocabulary. We especially checked the license restrictions of the vocabularies that do not permit their preparation for the OMOP CDM. Based on the search results from the first step, we summarized all codes, the hierarchy of the vocabulary, the corresponding German terms, and validity periods per vocabulary in tabular form for those vocabularies that do not have license restrictions. If no validity period was found, we used the default OMOP CDM values for *valid_start_date* (“1970-01-01”) and *valid_end_date* (“2099-12-31”) [14]. Next, we translated the German designations into English to make the semantic meaning of the codes understandable in an international context. Two researchers with domain knowledge were involved in the translations and their validation. For the fourth step of OMOP-compliant vocabulary preparation, there were 2 different methods available [15]: mapping to standard concepts using Usagi [16,17] and OMOP-compliant preparation of new 2-billion concepts (ie, *concept_id* >2 billion). Both methods are described in more detail in the following sections. Finally, we evaluated the results from the fourth step.

#### Mapping to Standard Concepts

To map local codes from a source system to standard concepts used in the OMOP CDM, the OHDSI community provides the open-source tool Usagi [16,17]. Usagi uses a term-similarity approach to propose appropriate standard concepts in the OMOP standardized vocabulary based on the English designation of the source codes. In the first step, we loaded a prepared list of source codes and their German and English designations into Usagi. Next, we specified the target domain of the OMOP standardized vocabulary that should be used during the mapping, based on the categories assigned to the German vocabularies shown in Figure 2. In a team of 2 researchers with domain knowledge, we reviewed the proposals made by Usagi and jointly discussed and resolved conflicts that had arisen during the mapping process. After the 2 researchers approved all proposals, the mapping of the source codes to standard concepts was exported as a CSV file following the format structure of the OMOP CDM *source_to_concept_map* table.

#### New 2-Billion Concepts

**Figure 4.**
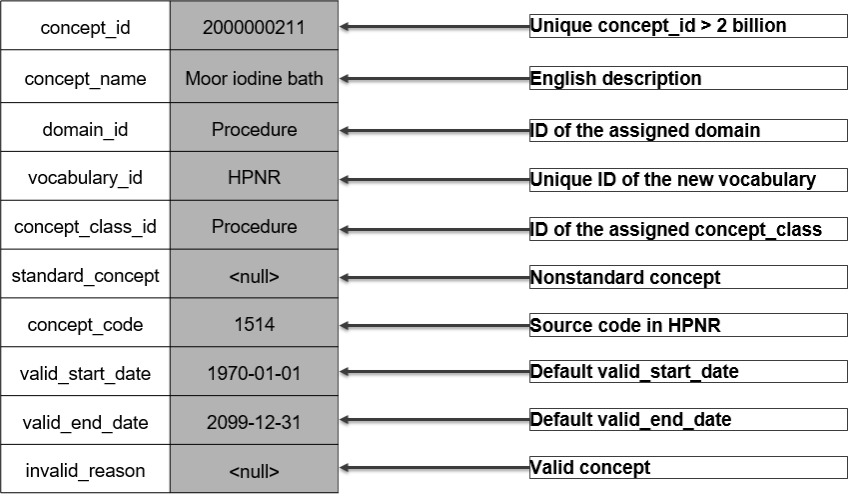
A new 2-billion concept in the Observational Medical Outcomes Partnership (OMOP) Common Data Model (CDM) *concept* table for HPNR source code ”1514.” HPNR: Heilmittelpositionsnummern (uniform list of item numbers for therapeutic services).

If source codes cannot be mapped using the standardized OMOP vocabularies, it is possible to create new 2-billion concepts for the OMOP CDM [15], which is a number range reserved for local *concept* creation. With this approach, we prepared the missing vocabularies to be OMOP compliant for the OMOP CDM tables *vocabulary*, *concept*, *concept_class*, and *concept_relationship*. Figure 4 shows the preparation of a new 2-billion concept for the OMOP CDM *concept* table for the *Heilmittelpositionsnummern* (HPNR; uniform list of item numbers for therapeutic services) source code “1514.”

During the preparation process, it must be taken into account that the new concepts are assigned a *concept_id* >2 billion to avoid conflicts with existing OMOP vocabularies [15]. For missing source codes, for example, for hierarchy levels, we used the prefix “OMOP” followed by number sequence as the *concept_code*, similar to the *concept_codes* of the OMOP CDM vocabulary OMOP Extension; this procedure follows the convention that a combination of *vocabulary_id* and *concept_code* is supposed to be unique and serves as a secondary key. Furthermore, we had to assign *domains* and *concept_classes* to the new concepts. This was done by searching the English designation of the parent categories of the source codes in Athena and comparing it with the proposed concepts. After a review process consisting of 2 researchers, we decided on a suitable *domain* and *concept_class* and added them to the parent categories as well as to the specific source codes. After the preparation of the *concept* table was completed, we stored the hierarchical structure of the vocabulary, as well as the information about replaced invalid concepts (eg, due to a change in the designation of a source code), in the *concept_relationship* table.

#### Evaluation

After the vocabulary preparation, we evaluated the mapping results from Usagi as well as the new 2-billion concepts regarding the completeness of the source codes and the correctness of the validity periods using the synthetic claims data. For both criteria, we implemented an evaluation process with Pentaho Data Integration (Hitachi Vantara) [18]. The input of this process was a list of source codes and the corresponding dates extracted from the synthetic claims data set, as well as the prepared *source_to_concept_map* or *concept* table as CSV files for each vocabulary. Consequently, no connection to an OMOP CDM database was required. Both evaluations were done by an interdisciplinary team in an iterative process until all identified errors had been resolved.

The aim of the completeness assessment was to check if all source codes are available in the prepared vocabularies. This was done by searching the source codes in the *source_code* (*source_to_concept_map*) or *concept_code* (*concept*) columns. If the result of the search had found a *concept_id*, then the codes were already present in the vocabulary; otherwise, they were missing. For both findings, the occurrence of the unique codes in the source record was then calculated and exported as CSV files as a result. In the next step, we further analyzed the missing codes—whether they were forgotten during preparation or if they displayed data quality issues—by using the vocabulary preparation approach for the missing codes, as illustrated in Figure 3.

The second evaluation focused on the correct assignment of the validity periods of the source codes in the prepared vocabularies. For this purpose, we used the source codes for which a matching *concept_id* was found during the first evaluation step. During the correctness assessment, we further checked whether the source codes with their associated dates were valid in the prepared vocabularies. The verification was based on a lookup to identify if the date used in the source was within the validity period of the vocabulary. A positive result indicated that codes were valid, and a negative result indicated that codes were invalid. For both results, we again calculated the occurrence of the unique combination of source code and date in the source and exported the results as CSV files. Afterward, we examined the invalid codes and rechecked whether the validity periods in the vocabularies contained errors.

### Final Determination of the Vocabulary Coverage

According to the approach used in Henke et al [12] to calculate the initial vocabulary coverage in the OMOP CDM, we recalculated the vocabulary coverage after the vocabulary preparation. For this purpose, we took our initial list of all German vocabularies used in the claims data set and assigned them again to 3 categories: “available in Athena,” “available through interim mapping,” and “not available.” With regard to the new 2-billion concepts, we added a new fourth category called “Athena-ready.” This category was intended to show that for the new 2-billion concepts, only the external step of loading them into Athena remains. During the final determination, a score of 1 was assigned to a vocabulary if it belongs to the category, and 0 was assigned otherwise. Based on the scores, we calculated the percentage distribution among the 4 categories.

## Results

### Vocabulary Preparation

Within the selective search, we were able to collect information for all of the missing vocabularies shown in Figure 2 (see Multimedia Appendix 1). However, due to license restrictions, 2 vocabularies (German Diagnosis Related Groups and *Pharmazentralnummer* [Central Pharmaceutical Number]) are legally not allowed to be prepared for the OMOP CDM. For the preparation of the other missing vocabularies, we applied both approaches by using Usagi to map the OMOP CDM standard concepts and by creating new 2-billion concepts. From Table 1, it can be seen that we applied a mapping to standard concepts for 3 vocabularies: *Ambulante spezialfachärztliche Versorgung* (ASV; outpatient specialist care), diagnosis type (inpatient), and provider specialty. These vocabularies were prepared with Usagi when a comprehensive mapping to OMOP CDM standard concepts was possible. The resulting Usagi export of the *source_to_concept_map* table for each vocabulary can be found in our GitHub repository [19].

**Table 1.** Overview of vocabularies prepared with Usagi, including number of records in the *source_to_concept_map* table.

Vocabulary name	*Source_to_concept_map*, records, n
ASV^a^	68
Diagnosis type (inpatient)	6
Provider specialty	104

a ASV: Ambulante spezialfachärztliche Versorgung (outpatient specialist care).

For the remaining 8 vocabularies, we created new 2-billion concepts to map the source codes to unique *concept_ids* that are analogous to other nonstandard concepts in the OMOP CDM and to prevent different semantic meanings of source codes according to the example shown in Figure 1. Table 2 provides an overview of the number of newly added records for the *vocabulary*, *concept_class*, *concept,* and *concept_relationship* tables for each new 2-billion concept (see the GitHub repository [20] for details). We have prepared almost all vocabularies entirely, that is, all source codes contained in the vocabularies. The exceptions are the *Einheitlicher Bewertungsmaßstab* (EBM; German Uniform Assessment Standard) and inpatient charge types vocabularies. In the case of EBM, we prepared the EBM vocabulary widely used in Germany for the OMOP CDM. For inpatient charge types, we restricted the preparation to the hierarchy levels “daily charges” and “case-related charges” that were relevant in the Hybrid-QI project.

**Table 2.** Overview of vocabularies prepared as new nonstandard concepts, including number of records in the *vocabulary*, *concept_class*, *concept,* and *concept_relationship* tables.

Vocabulary name	*Vocabulary*, records, n	*Concept_class*, records, n	*Concept*, records, n	*Concept_relationship*, records, n
HMK^a^	1	2	263	396
HPNR^b^	1	0	732	1344
PIA^c^	1	0	74	0
EBM^d^	1	2	3614	7348
Inpatient charge types	1	2	992	1997
Outpatient charge types (including EBM)	1	2	5647	11,280
Diagnosis type (outpatient)	0	0	4	0
Diagnosis type (inpatient)	0	0	2	0

a HMK: Heilmittelkatalog (catalog of physiotherapy, podological therapy, speech therapy, occupational therapy, and nutritional therapy).

b HPNR: Heilmittelpositionsnummern (uniform list of item numbers for therapeutic services).

c PIA: Bundeseinheitlicher Katalog für die Dokumentation der Leistungen der psychiatrischen Institutsambulanzen (standardized federal catalog for the documentation of services provided by psychiatric institutional outpatient clinics).

d EBM: Einheitlicher Bewertungsmaßstab (German Uniform Assessment Standard).

### Evaluation Outcomes

For both variants of the vocabulary preparation through the *source_to_concept_map* or *concept* table, we evaluated completeness and correctness. The evaluation process implemented for this purpose has been released as a GitHub repository [20]. The process includes the extraction of source code and date information from synthetic German claims data, as well as the completeness and correctness assessment for each vocabulary. The following sections demonstrate the final results of the evaluation after multiple iterations.

### Completeness

The results of the vocabulary completeness evaluation are summarized in Table 3. From the results, it can be seen that 44.3% (3288/7417) of the source codes can be found in the prepared concepts for the OMOP CDM. What stands out in the table are the EBM and ASV vocabularies. For the EBM vocabulary, 68.21% (4074/5973) of the unique codes were not found in the prepared concepts, that is, they had no *concept_id*, which resulted in 21.68% (1,422,808/6,563,865) of source records not having a *concept_id*. For the ASV vocabulary, no *concept_id* was found for 21.74% (5/23) of the unique codes. Consequently, 8.07% (44/545) of the source records did not have a *concept_id*. Furthermore, we summed up the number of unique codes and total records of the provider specialty vocabulary in Table 3. This is because the vocabulary occurred in 5 different source tables that include data about remedies, outpatient drug prescriptions, and contract medical care.

**Table 3.** Results of the vocabulary completeness evaluation.

Vocabulary name	Unique source codes without *concept_id*, n/N^a^ (%)	Records without *concept_id*, n/N^b^ (%)
HMK^c^	12/116 (10.34)	120/70,514 (0.17)
HPNR^d^	1/105 (0.95)	5/137,106 (0.0036)
PIA^e^	0/37 (0)	0/8721 (0)
EBM^f^	4074/5973 (68.21)	1,422,808/6,563,865 (21.68)
ASV^g^	5/23 (21.74)	44/545 (8.07)
Inpatient charge types	0/101 (0)	0/1222 (0)
Outpatient charge types	37/1062 (3.48)	1760/48,422 (3.63)
Diagnosis type (outpatient)	0/3 (0)	0/17,437 (0)
Diagnosis type (inpatient)	0/6 (0)	0/864,764 (0)
Provider specialty	0/104 (0)	0/9,057,555 (0)

a n=number of unique source codes with *concept_id*; N=number of unique source codes.

b n=number of records without *concept_id*; N=number of records.

c HMK: Heilmittelkatalog (catalog of physiotherapy, podological therapy, speech therapy, occupational therapy, and nutritional therapy).

d HPNR: Heilmittelpositionsnummern (uniform list of item numbers for therapeutic services).

e PIA: Bundeseinheitlicher Katalog für die Dokumentation der Leistungen der psychiatrischen Institutsambulanzen (standardized federal catalog for the documentation of services provided by psychiatric institutional outpatient clinics).

f EBM: Einheitlicher Bewertungsmaßstab (German Uniform Assessment Standard).

g ASV: Ambulante spezialfachärztliche Versorgung (outpatient specialist care).

### Correctness

The correctness evaluation was performed for the source codes for which a *concept_id* was found during the completeness evaluation. Furthermore, we excluded records if they had a missing source code or date information in the source. For the diagnosis type and provider specialty vocabularies, we were unable to evaluate the correctness due to having no equivalent dates in the source. In addition, these vocabularies have default values for *valid_start_date* and *valid_end_date* in the OMOP CDM. Table 4 shows the results obtained from the correctness evaluation. Looking at the percentage of invalid unique source code–date combinations, for all 7 vocabularies, less than 1% (501/706,032) of the combinations were invalid. In comparison, considering the total number of records, less than 1% (3036/5,358,996) of the records were identified as invalid. The vocabulary with the lowest correctness was HPNR with 0.13% (442/33,439) of invalid unique source code–date combinations and 2.14% (2938/137,101) of invalid records.

**Table 4.** Results of the vocabulary correctness evaluation.

Vocabulary Name	Invalid unique source code–date combination, n/N^a^ (%)	Invalid records, n/N^b^ (%)
HMK^c^	2/27,197 (0.0074)	2/70,394 (0.0028)
HPNR^d^	442/33,439 (0.13)	2938/137,101 (2.14)
PIA^e^	0/2449 (0)	0/8721 (0)
EBM^f^	56/641,285 (0.0087)	95/5,141,057 (0.0018)
ASV^g^	0/490 (0)	0/501(0)
Inpatient charge types	1/1172 (0.09)	1/1222 (0.08)
Outpatient charge types	0/39,123 (0)	0/46,662 (0)

a n=number of invalid unique source–date combinations; N=number of unique source–date combinations.

b n=number of invalid records; N=number of records.

c HMK: Heilmittelkatalog (catalog of physiotherapy, podological therapy, speech therapy, occupational therapy, and nutritional therapy).

d HPNR: Heilmittelpositionsnummern (uniform list of item numbers for therapeutic services).

e PIA: Bundeseinheitlicher Katalog für die Dokumentation der Leistungen der psychiatrischen Institutsambulanzen (standardized federal catalog for the documentation of services provided by psychiatric institutional outpatient clinics).

f EBM: Einheitlicher Bewertungsmaßstab (German Uniform Assessment Standard).

g ASV: Ambulante spezialfachärztliche Versorgung (outpatient specialist care).

### Vocabulary Coverage

After the evaluation of the prepared vocabularies, we checked the impact of the standard concept mappings and the new 2-billion concepts on the vocabulary coverage in the OMOP CDM. Table 5 shows the results of the final determination of the vocabulary coverage (see Multimedia Appendix 2). As can be seen, the percentage of unavailable vocabularies decreased noticeably from 55% (11/20) to 10% (2/20). One reason for this is the increase of interim mappings from 30% (6/20) to 45% (9/20) using the *source_to_concept_map* table. Another impact has been the creation of new 2-billion concepts for the OMOP CDM. With this approach, 30% (6/20) of the vocabularies were prepared for the OMOP CDM as Athena-ready concepts. The remaining 10% (2/20), which refers to the unavailable vocabularies, is because of the 2 missing vocabularies due to license restrictions.

**Table 5.** Vocabulary coverage of German claims data in the Observational Medical Outcomes Partnership (OMOP) Common Data Model (CDM) after vocabulary preparation.

Vocabulary coverage status	Vocabularies (N=20), n (%)
Available in Athena	3 (15)
Athena ready	6 (30)
Available through interim mapping	9 (45)
Not available	2 (10)

## Discussion

### Vocabulary Preparation Approach

With our presented approach of vocabulary preparation consisting of mapping to standard concepts and the manual creation of new 2-billion concepts, we could improve the vocabulary coverage of missing German vocabularies for use within the OMOP CDM. By preparing 10 vocabularies, we were able to show that our approach is applicable to any type of vocabulary used in a source data set. Referring to our objectives, we succeeded in mapping the majority of source codes used in German claims data to matching *concept_ids* in the OMOP CDM (objective 1). Furthermore, by mapping source codes to standard concepts and creating new 2-billion concepts in the OMOP CDM, we are now able to use German claims data for research based on the OMOP CDM (objective 2).

Nevertheless, there are limitations to our work. The first limitation relates to the reusability of the prepared vocabularies for other researchers. Our newly created 2-billion concepts are not currently available in Athena. Until the integration into Athena is done, the prepared vocabularies are available for other researchers via download from our GitHub repository [19]. When using our vocabularies in the OMOP CDM at other sites, it should be considered that conflicts with 2-billion concepts from other researchers must be avoided by agreeing on certain number ranges within the 2-billion range. A solution for this would be to set up a blank OMOP CDM database, where only the vocabularies provided by Athena are present and our prepared vocabularies are loaded afterward.

Furthermore, our results are currently limited to German vocabularies, which can only be used in national research projects using the OMOP CDM, because the mapping of new 2-billion concepts to standard concepts has not yet taken place. Consequently, the data cannot be used for international studies that are based on OMOP CDM standard concepts. To be able to participate in OHDSI network studies with German claims data, future work is required to map the prepared 2-billion concepts to standard concepts.

### Vocabulary Completeness

Our vocabulary completeness evaluation showed that we are currently covering 44.3% (3288/7417) of the codes used in the synthetic German claims data set. Nevertheless, even after multiple iterative adjustments of the vocabularies for missing source codes, some source codes could still not be found in the prepared vocabularies, especially for the Heilmittelkatalog (HMK; catalog of physiotherapy, podological therapy, speech therapy, occupational therapy, and nutritional therapy), HPNR, EBM, ASV, and outpatient charge types vocabularies. The reasons for missing codes can be divided into 2 categories: data quality issues and vocabulary scope limitation. The category of data quality issues summarizes causes such as transposed digits, missing characters, or coding errors (the number zero instead of the letter “O”). Thus, these errors do not refer to incomplete prepared vocabularies but to documentation mistakes in the source. For this reason, the errors are reflected back to the data-providing site, to serve as a basis for future work regarding the development of methods to increase the documentation quality, for example, through a preprocessing of data before they are loaded into the OMOP CDM.

The category of vocabulary scope limitation mainly refers to the EBM vocabulary. For our purpose, we prepared the EBM vocabulary widely used in Germany for the OMOP CDM. However, there also exist local codes administered by the regional associations of statutory health insurance physicians, which can serve as extensions or substitutions to the Germany-wide EBM. The preparation of local EBM codes will be a part of future work.

During the implementation of the evaluation process and the analysis of the results, we encountered challenges for the outpatient charge types vocabulary. The outpatient charge types vocabulary refers to a selection of EBM codes. For this reason, 2 prepared vocabularies had to be considered in the evaluation of completeness. However, for codes that could not be found in either of the 2 vocabularies, it was not possible to assess from which vocabulary they originated. In our next steps of semantic mapping of German claims data to the OMOP CDM, we plan to write such unassignable codes with a *concept_id* of 0 to the OMOP CDM to highlight this issue.

### Vocabulary Correctness

The results of the vocabulary correctness evaluation revealed invalid source codes for the HMK, HPNR, EBM, and inpatient charge types vocabularies. Looking at it in more detail, we found that both reasons for invalid codes, that is, invalid codes because of dates before the *valid_start_date* in the OMOP CDM and invalid codes because of dates after the *valid_end_date* in the OMOP CDM, occurred during our evaluation. Although the first problem occurred for all 4 vocabularies, the last 1 only occurred for the EBM vocabulary. A possible explanation for this might be the difference between the date of service provision and the date of payment in the source. Ditscheid et al [21] have already highlighted the temporal discrepancies of these 2 dates and their influence on the interpretation of results. They found that discrepancies in time could lead to an underestimation or overestimation of health service utilization regarding the death date of a patient or the change between years. They proposed to take these discrepancies “into account when requesting the data, but also in preparing and analyzing them” [21]. Consequently, when conducting research with (German) claims data based on the OMOP CDM, we must decide individually for each vocabulary which dates are appropriate for checking their validity in the OMOP CDM concept. However, since only a single piece of date information per vocabulary was available in the synthetic German claims data set, the evaluation conducted in this paper was limited to this information and should be repeated in the future in a second evaluation with more suitable date information per vocabulary.

### Comparison With Prior Work

Our proposed vocabulary preparation approach is consistent with methods used by other researchers. There are many papers describing the mapping of source codes or even free texts to OMOP CDM standard concepts using Usagi [8,13,22-25]. However, many papers also describe the approach of creating new 2-billion concepts, since mapping to standard concepts is not always possible [7,9,13,26-31]. For example, Fischer et al [28] created custom concepts for the Pulmonary Hypertension Nice classification. Rinner et al [29] focused on the missing vocabulary of the Austrian pharmaceutical registration number and consequently created new records for the *vocabulary*, *concept_class,* and *concept* tables in the OMOP CDM. Sathappan et al [13] created new unique 2-billion *concept_ids* to store local questionnaire terms in the OMOP CDM. However, none of these approaches describe in detail the process of preparing missing vocabularies for the OMOP CDM. The paper by Sathappan et al [13] laid out a promising approach. However, newly created concepts were directly added as standard concepts to the OMOP CDM, which limits their use to local analysis.

Compared to other research, our approach offers 2 advantages. In terms of a guideline, the presented approach enables the preparation of missing vocabularies for the OMOP CDM for a specific site’s data as well as their evaluation. Furthermore, making the vocabularies available via GitHub enables distribution and direct use of the newly created vocabularies for German data and, thus, ensures semantic interoperability across institutions in Germany.

### Conclusions

With our presented vocabulary preparation approach, we took a first promising step toward using German claims data for research based on the OMOP CDM. German health care providers, institutes, and health insurance companies can use the prepared German vocabularies, as these vocabularies are part of the legal data transmission for billing processes with health insurance companies. However, the proportion of newly created 2-billion concepts cannot yet be used for international studies due to a missing mapping to standard concepts. During our next steps, we will address this problem by using Usagi to propose a mapping from the new 2-billion concepts to standard concepts. By doing so, we also want to investigate how well the new German 2-billion concepts can be mapped to OMOP CDM standard concepts and identify the reasons why specific German codes could not be mapped (eg, missing semantic concepts or specifics of the German health care system [billing focus]). In addition, we also aim to collaborate with OHDSI to have our prepared vocabularies externally validated and subsequently integrated into Athena.

## Supplementary material

10.2196/47959 Multimedia Appendix 1 Overview of relevant links to the vocabularies considered during the preparation process.

10.2196/47959 Multimedia Appendix 2 Results of the final determination of vocabulary coverage.

## Abbreviations

ASV: Ambulante spezialfachärztliche Versorgung (outpatient specialist care)
CDM: common data model
EBM: Einheitlicher Bewertungsmaßstab (German Uniform Assessment Standard)
HMK: Heilmittelkatalog (catalog of physiotherapy, podological therapy, speech therapy, occupational therapy, and nutritional therapy)
HPNR: Heilmittelpositionsnummern (uniform list of item numbers for therapeutic services)
Hybrid-QI: Hybrid Quality Indicators Using Machine Learning Methods
*ICD-10-GM*: *International Classification of Diseases, Tenth Revision, German Edition*
OHDSI: Observational Health Data Sciences and Informatics
OMOP: Observational Medical Outcomes Partnership
RWD: real-world data
