# Supplementary material for: Assessing the Use of German Claims Data Vocabularies for Research in the Observational Medical Outcomes Partnership Common Data Model: Development and Evaluation Study
Source: JMIR Med Inform. 2023 Nov 7;11:e47959. doi: 10.2196/47959 (PMC10653283; doi:10.2196/47959)
Supplement: Multimedia Appendix 1 [file medinform-v11-e47959-s001.docx]

### Overview of relevant links to the vocabularies considered during the preparation process

#### HMK

- https://heilmittelkatalog.de/
- https://www.heilmittelkatalog.app/
- https://www.g-ba.de/richtlinien/12/historie/
- https://applications.kbv.de/S_HM_DIAGNOSEGRUPPE.xhtml
- https://simplifier.net/guide/eHeilmittelverordnung/CodeSystemfrdieDiagnosegruppe2?version=current

#### HPNR

- https://www.gkv-datenaustausch.de/leistungserbringer/sonstige_leistungserbringer/positionsnummernverzeichnisse/positionsnummernverzeichnisse_archiv.jsp

#### PIA

- Datenübermittlung nach § 301 Abs. 3 SGB V: Schlüssel 23 ‚PIA-Leistung’
- https://www.dkgev.de/themen/digitalisierung-daten/elektronische-datenuebermittlung/datenuebermittlung-zu-abrechnungszwecken/datenuebermittlung-nach-301-abs-3-sgb-v/

#### EBM

- Datenübermittlung nach § 301 Abs. 3 SGB V: Anhang D zu Anlage 2: ‚EBM-Ziffern‘
- https://www.dkgev.de/themen/digitalisierung-daten/elektronische-datenuebermittlung/datenuebermittlung-zu-abrechnungszwecken/datenuebermittlung-nach-301-abs-3-sgb-v/
- https://www.kbv.de/html/ebm.php

#### ASV

- Datenübermittlung nach § 301 Abs. 3 SGB V: Schlüssel 22 ‚Leistungsbereich (116b)‘
- https://www.dkgev.de/themen/digitalisierung-daten/elektronische-datenuebermittlung/datenuebermittlung-zu-abrechnungszwecken/datenuebermittlung-nach-301-abs-3-sgb-v/
- https://www.kbv.de/html/8160.php
- https://bv-asv.de/asv/indikationen/
- https://institut-ba.de/service/asvabrechnung.html

#### Inpatient charge types

- Datenübermittlung nach § 301 Abs. 3 SGB V: Schlüssel 4 ‚Entgeltarten’ Teil I: ‚Entgeltarten stationär‘
- Datenübermittlung nach § 301 Abs. 3 SGB V: Anhang B zu Anlage 2: ‚Entgeltarten (stationär)‘
- https://www.dkgev.de/themen/digitalisierung-daten/elektronische-datenuebermittlung/datenuebermittlung-zu-abrechnungszwecken/datenuebermittlung-nach-301-abs-3-sgb-v/
- https://kh-entgeltschluessel.gkv-datenaustausch.de/

#### Outpatient charge types

- Datenübermittlung nach § 301 Abs. 3 SGB V: Schlüssel 4 ‚Entgeltarten’ Teil II: ‚Entgeltarten ambulant‘
- Datenübermittlung nach § 301 Abs. 3 SGB V: Anhang B zu Anlage 2: ‚Entgeltarten (ambulant)‘
- https://www.dkgev.de/themen/digitalisierung-daten/elektronische-datenuebermittlung/datenuebermittlung-zu-abrechnungszwecken/datenuebermittlung-nach-301-abs-3-sgb-v/
- https://kh-entgeltschluessel.gkv-datenaustausch.de/

#### Diagnosis type (inpatient and outpatient)

- The diagnosis type vocabulary (codes and their designation) were part of the synthetic German claims dataset.

#### Provider specialty

- The provider specialty vocabulary (codes and their designation) were part of the synthetic German claims dataset.
